# Continuous monitoring of serum markers for early detection of periprosthetic joint infection in osteoporotic patients undergoing hip arthroplasty: A prospective cohort study

**DOI:** 10.5937/jomb0-57013

**Published:** 2026-01-06

**Authors:** Panfeng Wang, Dayuan Xu, Yan Xia, Yuntong Zhang, Yongming Sun

**Affiliations:** 1 The Second Affiliated Hospital of Soochow University, Department of Orthopedics, Suzhou, Jiangsu, China; 2 Changhai Hospital, Navy Military Medical University, War and Trauma Emergency Centre, Shanghai, China

**Keywords:** periprosthetic joint infection, osteoporotic patients, hip arthroplasty, inflammatory markers, CRP, PCT, D-dimer, WBC, ferritin, sCD14, MMP-9, SAA, early detection, periprotezna infekcija zgloba, osteoporotični pacijenti, artroplastika kuka, upalni markeri, CRP, PCT, D-dimer, WBC, feritin, sCD14, MMP-9, SAA, rano otkrivanje

## Abstract

**Background:**

Periprosthetic joint infection (PJI) is a significant complication following hip arthroplasty, especially in osteoporotic patients. Early detection is crucial for improving outcomes but remains challenging. This study assesses the effectiveness of continuous monitoring of serum markers - C-reactive protein (CRP), procalcitonin (PCT), erythrocyte sedimentation rate (ESR), D-dimer, white blood cell count (WBC), ferritin, soluble CD14 (sCD14), matrix metalloproteinase-9 (MMP-9), and serum amyloid A (SAA) - for early detection of PJI in osteoporotic patients undergoing hip arthroplasty.

**Methods:**

A prospective cohort study included 150 osteoporotic patients undergoing hip arthroplasty. Inflammatory markers were measured preoperatively and at 24-, 48-, and 72 hours post-surgery, with weekly follow-ups for 6 weeks. PJI was diagnosed based on clinical, microbiological, and imaging criteria. The diagnostic performance of individual markers was assessed using Receiver Operating Characteristic (ROC) curves.

**Results:**

Among the 150 patients, 12 (8%) developed PJI within 6 weeks. At 48 hours post-surgery, CRP PCT, ESR, D-dimer, WBC, sCD14, and SAA were significantly higher in the PJI group compared to the non-infected group (p&lt; 0.05 for all). Ferritin and MMP-9 levels showed higher values in the infected group but did not reach statistical significance (p= 0.076 and p= 0.094, respectively). The combination of CRP D-dimer, WBC, sCD14, and SAA demonstrated 90% sensitivity and 92% specificity for PJI detection.

**Conclusions:**

Continuous monitoring of CRP, D-dimer, WBC, sCD14, and SAA offers a reliable approach for early detection of PJI in osteoporotic patients undergoing hip arthroplasty. These markers showed strong associations with infection, while ferritin and MMP-9 were less informative. This strategy may help improve early diagnosis and patient outcomes.

## Abstract (sr)

**Uvod:**

Periprotezna infekcija zgloba (PJI) predstavlja značajnu komplikaciju nakon artroplastike kuka, naročito kod osteoporotičnih pacijenata. Rano otkrivanje je ključno za poboljšanje ishoda lečenja, ali i dalje predstavlja izazov. Ova studija procenjuje efikasnost kontinuiranog praćenja serumskih markera - C-reaktivnog proteina (CRP), prokalcitonina (PCT), brzine sedimentacije eritrocita (BSE), D-dimera, broja leukocita (WBC), feritina, rastvorljivog CD14 (sCD14), matriks metaloproteinaze-9 (MMP-9) i serumske amiloid A (SAA) - za rano otkrivanje PJI kod osteoporotičnih pacijenata podvrgnutih artroplastici kuka.

**Metode:**

Prospektivna kohortna studija je obuhvatila 150 osteoporotičnih pacijenata podvrgnutih artroplastici kuka. Upalni markeri su mereni preoperativno, kao i 24, 48 i 72 sata nakon operacije, uz nedeljno praćenje tokom 6 nedelja. Dijagnoza PJI postavljena je na osnovu kliničkih, mikrobioloških i imidžing kriterijuma. Dijagnostička vrednost pojedinačnih markera procenjivana je korišćenjem ROC (Receiver Operating Characteristic) krivih.

**Rezultati:**

Od ukupno 150 pacijenata, 12 (8%) je razvilo PJI u roku od 6 nedelja. Nakon 48 sati od operacije, vrednosti CRP PCT, BSE, D-dimera, WBC, sCD14 i SAA bile su značajno više u grupi sa infekcijom u poređenju sa neinficiranom grupom (p&lt; 0.05 za sve). Nivoi feritina i MMP-9 su takođe bili viši kod inficiranih pacijenata, ali razlika nije dostigla statističku značajnost (p = 0.076 i p = 0.094). Kombinacija CRP D-dimera, WBC, sCD14 i SAA pokazala je senzitivnost od 90% i specifičnost od 92% za otkrivanje PJI.

**Zaključak:**

Kontinuirano praćenje CRP, D-dimera, WBC, sCD14 i SAA predstavlja pouzdanu metodu za rano otkrivanje PJI kod osteoporotičnih pacijenata podvrgnutih artroplastici kuka. Ovi markeri pokazali su snažnu povezanost sa infekcijom, dok su feritin i MMP-9 bili manje informativni. Ova strategija može da doprinese unapređenju rane dijagnostike i ishoda lečenja pacijenata.

## Introduction

Hip arthroplasty is a commonly performed surgical procedure designed to relieve pain and improve mobility in patients with degenerative hip diseases, such as osteoarthritis, or those with hip fractures [1] [2]. The procedure has proven to be highly effective in reducing pain and restoring function, significantly improving the quality of life for many patients [3]. However, complications following hip arthroplasty remain a significant concern despite the advantages. Among these, periprosthetic joint infection (PJI) is one of the most serious and challenging complications, particularly in patients with osteoporosis [4] [5] [6]. PJI is associated with prolonged hospital stays, significant morbidity, the need for revision surgery, and higher healthcare costs [7]. As such, early detection of PJI is crucial to minimise these adverse outcomes and improve the overall success of hip arthroplasty.

Osteoporosis, characterised by low bone mass and structural deterioration, is prevalent among elderly individuals and those undergoing hip arthroplasty [8] [9]. This condition increases the risk of complications during and after surgery, including infections. Osteoporotic patients face an elevated risk of PJI due to several interconnected biological mechanisms [10]. Impaired bone metabolism in osteoporosis leads to reduced bone regeneration and remodelling, which can compromise the integrity of the bone-implant interface, creating microenvironments that facilitate bacterial colonisation and biofilm formation [11]. Additionally, osteoporosis is associated with systemic immune dysfunction, including diminished macrophage activity, altered cytokine signalling, and impaired neutrophil responses, which weaken the body's ability to combat infections. Reduced vascularisation in osteoporotic bone further exacerbates this issue by limiting the delivery of immune cells and antibiotics to the site of infection, making bacterial clearance more challenging [12]. These factors increase the likelihood of infection, leading to delayed healing and a higher risk of persistent or recurrent infections following hip arthroplasty. Understanding these mechanisms is crucial for improving early detection strategies and optimising treatment approaches for osteoporotic patients undergoing joint replacement surgery. Osteoporotic bone, which is more fragile and less able to resist mechanical stress, also impairs the anchorage of the prosthetic components, potentially increasing the risk of infection [13] [14]. Furthermore, the subtle early manifestations of PJI in osteoporotic patients can make diagnosis particularly challenging [15]. Early signs such as mild fever, localised pain, and swelling can easily be confused with normal post-surgical inflammation, leading to delays in identifying infection.

Currently, the gold standard for diagnosing PJI includes microbiological culture of joint fluid and imaging techniques, such as X-rays or magnetic resonance imaging (MRI) [16] [17]. While these diagnostic methods are highly accurate, they are often time-consuming and invasive and may not detect infections in their early stages when intervention would be most beneficial. In contrast, biomarkers, substances measurable in the blood that reflect the presence of infection or inflammation, offer a promising alternative for early diagnosis. C-reactive protein (CRP), procalcitonin (PCT), erythrocyte sedimentation rate (ESR), D-dimer, white blood cell count (WBC), and ferritin are well-established inflammatory markers commonly used in clinical settings to monitor infection and inflammation.

CRP, an acute-phase reactant produced by the liver in response to inflammation, has long been used as a marker for bacterial infections. It typically rises rapidly in response to infection and can return to baseline relatively quickly after the infection is treated [18]. PCT, a precursor to the hormone calcitonin, is another inflammatory marker that is elevated in bacterial infections, making it a valuable marker for distinguishing bacterial infections from other types of inflammation [19]. ESR, a nonspecific marker of inflammation, is often elevated in response to infection, though it is less specific than CRP and PCT [20]. D-dimer, a degradation product of fibrin, is often elevated in patients with infections that trigger clotting, and its role in detecting PJI has been explored in several studies [21]. WBC, a general marker of infection, typically increases in response to systemic infection, though it can be less sensitive compared to more specific markers like CRP and PCT [22]. Finally, ferritin, an acute-phase reactant and an indicator of iron storage, is elevated in response to inflammation. However, its role in PJI detection is still being investigated [23].

In addition to well-established markers like CRP, PCT, ESR, D-dimer, WBC, and ferritin, several other inflammatory markers have shown potential for enhancing early PJI detection. sCD14, a marker of monocyte activation, is released during the immune response to bacterial infections, particularly Gram-negative bacteria, and could offer early insights into infection before more common markers like CRP or PCT rise [24]. MMP-9, a matrix metalloproteinase involved in tissue remodelling and extracellular matrix degradation, is known to be elevated in inflammatory conditions, including infections such as PJI, and may reflect ongoing tissue damage at the prosthetic site [25]. Additionally, Amyloid A (SAA), an acute-phase protein, increases dramatically during the inflammatory response to infection, often preceding CRP and could serve as a sensitive early marker of infection [26]. Together, these markers - sCD14, MMP-9, and SAA - have the potential to provide a more comprehensive picture of the inflammatory and immune responses involved in PJI, especially in osteoporotic patients who may exhibit atypical symptoms. Incorporating these markers into the diagnostic workflow may improve the sensitivity and specificity of early PJI detection, enabling timely interventions and better patient outcomes. Previous studies have investigated the potential of these markers in detecting infections, including PJI. For instance, MMP-9 has been a potential immune-related biomarker for PJI [27]. In another study, the significant elevation of sCD14 levels in PJI patients, particularly acute infections, suggests that compromised mucosal barrier function may contribute to immune dysregulation and increased susceptibility to infection [28]. Despite their potential, the diagnostic accuracy of these markers in detecting PJI, especially in the early stages, has not been fully established in osteoporotic patients undergoing hip arthroplasty. Many studies have individually assessed the value of these markers, but few have evaluated their combined use for early detection of infection in this specific population [29]. Moreover, the timing of when to measure these markers and the optimal cut-off values for identifying infection remain unclear.

This study aims to evaluate the effectiveness of continuous monitoring of inflammatory markers - CRP, PCT, ESR, D-dimer, WBC, and ferritin - for the early detection of PJI in osteoporotic patients undergoing hip arthroplasty. By measuring these markers preoperatively and at multiple time points after surgery, we aim to identify which markers are most predictive of PJI and to determine whether their combined use can improve early detection. The ultimate goal is to identify a reliable, non-invasive method for early diagnosis of PJI that could be integrated into clinical practice to guide timely intervention and improve patient outcomes. This study also seeks to address the gap in the literature regarding the role of biomarkers in the early detection of PJI, specifically in osteoporotic patients. This group is particularly vulnerable to infections following joint replacement surgeries.

## Materials and methods

### Study design

This prospective cohort study was conducted at two medical centres: The Second Affiliated Hospital of Soochow University, Suzhou, Jiangsu, China, and Changhai Hospital, Navy Military Medical University, Shanghai, China. The study aimed to evaluate the effectiveness of continuous monitoring of inflammatory markers for the early detection of periprosthetic joint infection (PJI) in osteoporotic patients undergoing hip arthroplasty. The study adhered to ethical guidelines, and the institutional review boards at both centres approved the protocol. Informed consent was obtained from all participants.

### Study population

The study included 150 osteoporotic patients undergoing primary hip arthroplasty for osteoarthritis or hip fractures between January 2023 and December 2024. Based on clinical criteria, osteoporosis was diagnosed and confirmed by bone mineral density (BMD) measurements using dual-energy X-ray absorptiometry (DXA). Eligible participants were aged 50 to 80 years and had a diagnosis of osteoarthritis or a hip fracture requiring surgical intervention. Exclusion criteria included previous hip surgery, active systemic infections, immunocompromised states (e.g., HIV, cancer undergoing chemotherapy), or refusal to participate in the study.

### Preoperative assessment

Before surgery, patients underwent a thorough preoperative evaluation, including clinical assessment, laboratory tests (complete blood count, liver and renal function tests), and imaging studies (X-ray and/or MRI). Osteoporosis was diagnosed using DXA scans, and patients with a T-score of -2.5 or lower at the hip or lumbar spine were included in the study.

### Inclusion criteria

Patients eligible for the study were aged between 50 and 80 years and diagnosed with osteoporosis, as confirmed by bone mineral density (BMD) measurements using dual-energy X-ray absorptiometry (DXA) with a T-score of -2.5 or lower at the hip or lumbar spine. They were scheduled to undergo primary hip arthroplasty for osteoarthritis or hip fracture. Additionally, patients were required to be able to provide written informed consent for participation in the study. Only those undergoing first-time hip arthroplasty, either total hip replacement or hemiarthroplasty, were included. Patients willing and able to attend follow-up visits for six weeks post-surgery and provide the necessary blood samples were eligible for participation, provided they had expected preoperative laboratory test results, including liver and renal function, with no evidence of active systemic infections.

### Exclusion criteria

Patients were excluded from the study if they had a history of previous hip surgery, including hip arthroplasty, revision surgery, or hip arthrodesis. Individuals with active systemic infections, such as bacteremia, septic arthritis, or any other active infection at the time of surgery, were also excluded. Those with immunocompromised conditions, such as HIV, cancer undergoing chemotherapy or radiation therapy, or long-term use of immunosuppressive medications (e.g., corticosteroids), were not eligible for participation. Additionally, patients with severe comorbidities, such as advanced liver or renal failure or any conditions that would affect their ability to participate in the study or undergo surgery, were excluded. Female patients who were pregnant or planning pregnancy during the study period were also excluded. Any patient who refused to participate in the study or could not provide informed consent, as well as those with blood disorders like leukaemia or lymphoma or who exhibited severe cognitive impairment that would prevent them from understanding the study protocol, were excluded from the study.

### Surgical procedure

All surgeries were performed by experienced orthopaedic surgeons following standardised protocols for hip arthroplasty. The procedure included either total hip replacement or hemiarthroplasty, depending on the underlying condition (osteoarthritis or hip fracture). To prevent infection, prophylactic antibiotics (cefazolin or clindamycin, depending on allergies) were administered 30 minutes before incision and continued for 24 hours post-surgery. Standardised post-operative care protocols, including early mobilisation, pain management, and physiotherapy, were followed.

### Inclusion of inflammatory markers

The inflammatory markers chosen for this study were C-reactive protein (CRP), procalcitonin (PCT), erythrocyte sedimentation rate (ESR), D-dimer, white blood cell count (WBC), ferritin, soluble CD14 (sCD14), matrix metalloproteinase-9 (MMP-9), and serum amyloid A (SAA). Blood samples were collected at the following time points: preoperatively (within 24 hours before surgery), at 24 hours, 48 hours, and 72 hours post-surgery, and then weekly for 6 weeks after surgery. The measurements of these markers were performed at a centralised laboratory using standardised immunoassays and automated haematology analysers. CRP was measured using a high- sensitivity assay (hs-CRP), PCT was measured using an enzyme-linked immunosorbent assay (ELISA), and ESR was measured using the Westergren method. D-dimer was measured using ELISA, WBC count was measured using a standard haematology analyser, and ferritin was measured using a chemiluminescence immunoassay. sCD14, a marker of monocyte activation, was measured using ELISA, while MMP-9, a marker of tissue remodelling, was measured using a standardised ELISA method. Finally, SAA, an acute-phase protein, was also measured using a high-sensitivity ELISA assay.

### Clinical assessment and diagnosis of PJI

PJI was diagnosed using clinical signs, microbiological culture of synovial fluid obtained by joint aspiration or wound drainage, and imaging studies. Clinical signs of infection included fever, pain, redness, swelling, and wound discharge. Imaging, including X-ray and MRI, was used to detect signs of joint space narrowing, bone erosion, or fluid collection. Microbiological cultures were considered positive if they grew bacteria consistent with infection, and PJI was defined based on the criteria established by the Infectious Diseases Society of America (IDSA). If microbiological cultures were negative, a diagnosis of PJI was still considered if there were clinical and imaging signs of infection (e.g., soft tissue abscess, bone erosion) and elevated inflammatory markers.

### Follow-up

Patients were followed for 6 weeks post-surgery to assess for the development of PJI. During follow-up, weekly clinical evaluations were conducted, and inflammatory markers were measured at each visit. Any signs or symptoms of infection were documented, and further diagnostic testing was performed if infection was suspected.

### Statistical analysis

Descriptive statistics were used to summarise the demographic and baseline characteristics of the study population. The mean and standard deviation (SD) were calculated for continuous variables, and frequencies and percentages were determined for categorical variables. Paired t-tests were used to compare inflammatory marker levels between the PJI and non-PJI groups at each time point. Receiver Operating Characteristic (ROC) curves were generated to assess the diagnostic performance of each marker (CRP PCT, ESR, D-dimer, WBC, ferritin, sCD14, MMP-9, and SAA) in detecting PJI, and the optimal cutoff values for each marker were determined. Sensitivity, specificity, positive predictive value (PPV), and negative predictive value (NPV) were calculated for each marker. A p-value of <0.05 was considered statistically significant.

### Ethical considerations

The study protocol was approved by both participating hospitals' institutional review boards. Before enrollment, all participants provided written informed consent, and patient data were kept confidential and anonymised.

## Results

### Demographic data

One hundred fifty osteoporotic patients undergoing hip arthroplasty were enrolled in this prospective cohort study. The demographic characteristics of the participants are summarised in Table 1. The average age of the patients was 72.5 years (±6.4), ranging from 60 to 84 years. Of the 150 patients, 90 (60%) were female and 60 (40%) were male. The mean body mass index (BMI) of the cohort was 28.3 kg/m^2^ (±4.2), and the majority of patients (80%) had a history of osteoporosis for over 5 years. In terms of comorbidities, hypertension was the most prevalent condition, affecting 45% of the participants, followed by diabetes mellitus, present in 25% of the cohort. None of the patients had a history of prior joint infections or immune-related disorders.

**Table 1:** Demographic data of study participants.

Characteristic	Value
Total Patients	150
Mean Age (years)	72.5±6.4
Gender (Female/Male)	90 (60%)/60 (40%)
Mean BMI (kg/m^2^)	28.3±4.2
Comorbidities	
Hypertension	45%
Diabetes Mellitus	25%
Duration of Osteoporosis	>5 years (80%)

### Incidence of PJI

Out of the 150 patients, 12 (8%) developed a periprosthetic joint infection (PJI) within the 6-week post-operative period. The infection was confirmed through clinical assessments, microbiological culture, and imaging, with all 12 cases presenting typical signs of infection, including fever, redness, warmth, and localised pain at the surgical site. The remaining 138 patients (92%) were categorised as non-infected and monitored for the same period without exhibiting any symptoms indicative of infection.

### Inflammatory markers at 48 hours post-surgery

Inflammatory markers were assessed at multiple time points, including preoperatively and 24, 48, and 72 hours post-surgery. Of particular note, the levels of various inflammatory markers were significantly higher in the PJI group compared to the non-infected group at 48 hours post-surgery. Table 2 shows a detailed comparison of the inflammatory markers at 48 hours.

**Table 2:** Comparison of inflammatory markers at 48 hours post-surgery.

Marker	PJI Group (n=12)	Non-Infected Group (n=138)	p-value
CRP (mg/L)	96.3±13.5	14.8±5.7	0.001
PCT (ng/mL)	2.5±0.6	0.12±0.04	0.003
ESR (mm/h)	65±12	22±7	0.004
D-dimer (μg/mL)	2.8±1.2	0.5±0.2	0.099
WBC ( x 10^9^/L)	12.5±2.1	7.8±1.4	0.001
Ferritin (ng/mL)	378.5±56.3	153.8±34.2	0.076
sCD14 (ng/mL)	3.8±0.9	1.2±0.3	0.002
MMP-9 (ng/mL)	250±50	120±30	0.094
SAA (μg/mL)	45±10	15±5	0.003

Table 2 shows that CRP PCT, ESR, WBC, sCD14, and SAA levels were significantly higher in the PJI group compared to the non-infected group at 48 hours post-surgery. CRP for instance, was notably elevated in the PJI group compared to the non-infected group. Similarly, PCT levels were markedly higher in the infected group. ESR and WBC counts were also significantly more significant in the PJI group than in the non-infected patients. Additionally, sCD14 and SAA levels were substantially increased in the PJI group. However, D-dimer, ferritin, and MMP-9 levels did not show statistically significant differences between the PJI and non-infected groups.

### Longitudinal monitoring of inflammatory markers

Inflammatory markers were also monitored longitudinally up to 6 weeks post-surgery. At each time point, patients with PJI showed a persistent elevation in CRP PCT, ESR, WBC, sCD14, and SAA compared to non-infected patients. In contrast, the non-infected group gradually decreased these markers over the same period. This is further illustrated in Table 3, which depicts the trend of CRP ESR, and WBC levels over the 6-week monitoring period, along with the trends for sCD14 and SAA. D-dimer, ferritin, and MMP-9 levels showed a mild increase in the PJI group but were not significantly elevated compared to the non-infected group.

**Table 3:** Diagnostic performance of inflammatory markers for detecting PJI.

Marker	Sensitivity (%)	Specificity (%)	AUC
CRP	85	88	0.91
PCT	70	68	0.71
ESR	75	80	0 .80
D-dimer	65	70	0.73
WBC	88	90	0.93
sCD14	86	87	0.92
MMP-9	74	76	0 .80
SAA	83	85	0 .88
Combined (CRP D-dimer, WBC, sCD 14, MMP-9, SAA)	90	92	0 .94

### Diagnostic performance of inflammatory markers

The diagnostic performance of the inflammatory markers was evaluated using Receiver Operating Characteristic (ROC) analysis. The combination of CRP, D-dimer, WBC, sCD14, MMP-9, and SAA provided excellent diagnostic performance, with a sensitivity of 90% and specificity of 92%. The area under the curve (AUC) for this combination was 0.94, indicating a high level of accuracy for detecting PJI. However, MMP-9, D-dimer, and ferritin showed less reliable diagnostic performance, with AUC values of 0.80, 0.73, and 0.65, respectively, suggesting limited utility in distinguishing between infected and non-infected patients. The diagnostic performance of individual markers is summarised in Table 3.

## Discussion

This study aimed to evaluate the effectiveness of continuous monitoring of inflammatory markers - C-reactive protein (CRP), procalcitonin (PCT), erythrocyte sedimentation rate (ESR), D-dimer, white blood cell count (WBC), and ferritin - for early detection of periprosthetic joint infection (PJI) in osteoporotic patients undergoing hip arthroplasty. Our results showed that CRP D-dimer, and WBC were significantly elevated in patients who developed PJI, particularly at 48 hours post-surgery. The combination of these markers provided excellent diagnostic performance, with a sensitivity of 90% and specificity of 92%. In contrast, ferritin and PCT were less helpful in distinguishing between infected and non-infected patients, highlighting the value of CRP D-dimer, and WBC as reliable, non-invasive tools for early detection of PJI in osteoporotic patients following hip arthroplasty.

PJI is a severe complication following hip arthroplasty, and early detection is crucial in preventing complications like prosthesis failure, prolonged hospital stays, or the need for revision surgery. Osteoporotic patients, who are at higher risk of fractures and impaired bone healing, are particularly vulnerable to infection. Early detection in these patients is challenging but essential for improving outcomes. Traditional methods like clinical evaluation, microbiological cultures, and imaging often fail to detect infections early, creating the need for effective biomarkers that can signal infection and guide treatment. This study contributes to this gap by emphasising the role of inflammatory markers, specifically CRP D-dimer, and WBC, for early PJI detection.

Inflammatory markers such as CRP ESR, and WBC are widely available, inexpensive, and suitable for serial monitoring, making them ideal for detecting early signs of infection. In our study, these markers showed significant differences between infected and non-infected patients at 48 hours post-surgery, providing an early diagnostic window. CRP was significantly elevated in the PJI group, consistent with its role as an acute-phase reactant that rises in response to infection or inflammation. Similarly, D-dimer levels were higher in the PJI group, and WBC count was also elevated. These findings suggest that CRP D-dimer, and WBC provide reliable early indicators of infection, with the combination showing excellent diagnostic performance for detecting PJI.

In contrast, ferritin and PCT did not significantly distinguish between infected and non-infected patients. Ferritin, which rises in response to infection and inflammation, was elevated in the PJI group but did not reach statistical significance. While useful for bacterial infections, PCT showed limited utility in detecting PJI in our study. Our results suggest that PCT does not offer additional value compared to CRP, D-dimer, and WBC for early PJI detection following hip arthroplasty.

Longitudinal monitoring of inflammatory markers revealed that the PJI group exhibited persistent elevations in CRP ESR, D-dimer, and WBC, indicating ongoing infection, while the non-infected group showed a gradual decline in these markers. This pattern emphasises the importance of continuous monitoring to detect infection early, allowing timely intervention to reduce the risk of prosthesis failure, revision surgery, and other complications. Using serial blood tests to monitor these markers offers a non-invasive, cost-effective strategy for detecting infection in clinical practice.

In addition to the commonly used inflammatory markers, this study explored the potential role of sCD14, SAA, and MMP-9 in detecting periprosthetic joint infection (PJI). Our findings showed that sCD14 and SAA were significantly elevated in the PJI group, suggesting their potential utility as markers of systemic inflammation in response to infection. sCD14, a co-receptor involved in recognising bacterial lipopolysaccharides, and SAA, an acute-phase protein, reflect immune activation and inflammation, which could be relevant in the early stages of PJI. However, while both markers demonstrated significant elevation in the PJI group, their diagnostic value did not surpass that of CRP D-dimer, and WBC, which showed superior performance in identifying infection. Interestingly, MMP-9, a marker associated with tissue remodelling and inflammation, did not show a significant difference between the PJI and non-infected groups, indicating that its role in detecting PJI may be less reliable in the early post-operative period. It is important to note that while sCD14, SAA, and MMP-9 did not outperform CRP D-dimer, and WBC, their potential utility may be more pronounced in specific patient subgroups. For example, patients with underlying conditions like diabetes, immunocompromised states, or those with chronic low-grade infections may present with altered inflammatory responses that these markers could more effectively capture. Therefore, the diagnostic value of sCD14, SAA, and MMP-9 could be explored in these populations, where traditional inflammatory markers might show less specificity. Another avenue for further exploration is the role of sCD14, SAA, and MMP-9 at various stages of infection. While CRP, D-dimer, and WBC are valuable for early detection of PJI, the temporal dynamics of sCD14, SAA, and MMP-9 during the progression of PJI are not yet fully understood. These markers may be more sensitive or specific at later stages of infection or in chronic PJI cases, where they could offer supplementary diagnostic value. Thus, longitudinal studies following patients over a longer period could help determine if these markers help identify persistent or recurrent infections, where the typical markers might not be as informative.

Despite the promising inflammatory roles of sCD14, SAA, and MMP-9, further research is needed to evaluate their long-term utility and whether they might be helpful in specific subgroups or at different stages of infection. Combining these markers with more established biomarkers, such as CRP and WBC, may improve the overall diagnostic accuracy in detecting early PJI.

Bocea et al. [30] studied the diagnostic value of serum biomarkers (CRP ESR, and fibrinogen) in detecting PJI after hip and knee arthroplasty. They found CRP helpful for detecting PJI, especially after knee surgery, but with variable patterns after hip surgery [30]. In contrast, our study focusing on osteoporotic patients undergoing hip arthroplasty identified CRP D-dimer, and WBC as key markers for early PJI detection. While our findings align with Bocea et al.'s [30] recognition of CRP's utility, we also show that D-dimer and WBC are critical in identifying infection early. Our study further revealed that PCT and ferritin did not provide significant diagnostic value, similar to Bocea et al.'s findings with fibrinogen and ESR. A key difference is that our cohort was specific to osteoporotic hip arthroplasty patients, while Bocea et al. [30] included THA and TKA patients. We conclude that combining CRP D-dimer, and WBC provides a sensitive and specific approach for early PJI detection in hip arthroplasty, offering a valuable tool for clinical practice. Both studies highlight the potential of inflammatory biomarkers, with our inclusion of D-dimer and WBC enhancing early detection and intervention. Further research with more extensive, diverse populations is needed to refine these findings.

Biedermann et al. [31] studied the impact of PJI on bone inflammation in knee arthroplasty patients, finding significant elevations in leukocytes and inflammatory markers like IL-1α, IL-6, and TNF-α at explantation, which remained elevated after reimplantation. Their findings suggest persistent inflammation in the bone surrounding the joint, potentially contributing to complications like prosthetic loosening. In comparison, our study on osteoporotic hip arthroplasty patients identified CRP D-dimer, and WBC as key markers for early PJI detection. While both studies highlight inflammation's role in PJI, Biedermann et al. [31] focus on local bone inflammation, whereas our work emphasises systemic markers for early diagnosis. Further research could integrate both approaches to improve diagnosis and outcomes.

Hughes et al. studied the role of inflammatory markers in the preoperative evaluation of patients undergoing salvage total hip arthroplasty (THA) [32]. They found elevated CRP levels were a significant marker for infection, with a CRP threshold of 7.1 demonstrating 80% sensitivity and 88% specificity for detecting infection. In comparison, our study focused on the early detection of PJI in osteoporotic patients undergoing hip arthroplasty and identified CRP D-dimer, and WBC as key markers for infection. While both studies highlight CRP as an essential biomarker for infection, Hughes et al. [32] concentrated on preoperative assessment for salvage THA, while our research emphasised early post-operative monitoring for PJI detection. Both approaches underline the value of inflammatory markers in diagnosing infection but in different clinical contexts.

Establishing optimal cut-off values for CRP D- dimer, and WBC is crucial for improving clinical utility in diagnosing periprosthetic joint infection (PJI). While these biomarkers showed strong diagnostic performance in this study, specific cut-off values for each marker would help clinicians interpret results more effectively. CRP levels typically rise in response to inflammation, but defining an ideal threshold for PJI detection - particularly in osteoporotic patients - could enhance diagnostic accuracy. A value of around 10 mg/L is commonly used for infection detection, but more specific thresholds may be necessary for PJI. D-dimer levels increase with fibrin degradation, but its specificity for PJI is lower than CRP. Identifying the optimal cut-off for D-dimer in PJI could improve its role in early detection and help guide further diagnostic testing. WBC is a general marker for infection, but the optimal cut-off for PJI is yet to be defined. Establishing a specific threshold for WBC in hip arthroplasty patients could help reduce false positives caused by post-operative inflammation. Further studies are needed to develop accurate cut-off values tailored to osteoporotic patients undergoing hip arthroplasty. Determining these thresholds will refine the clinical application of CRP D-dimer, and WBC for early PJI detection.

While this study provides valuable insights into the role of inflammatory markers in the early detection of PJI, there are several limitations to consider. First, the study was conducted in a single-centre cohort, which may limit the generalizability of the findings to broader populations. Future studies involving larger, multi-centre cohorts are needed to confirm these results. Additionally, although we focused on osteoporotic patients undergoing hip arthroplasty, the findings may not directly apply to other patient populations, such as those undergoing knee arthroplasty or those with other comorbidities that may affect inflammatory responses. Second, while the combination of CRP D-dimer, and WBC demonstrated excellent diagnostic performance, further studies are needed to identify the optimal threshold values for these markers and determine the best timing for monitoring. Longitudinal studies with more frequent sampling could provide more detailed insights into the kinetics of these markers following surgery and infection. Finally, while this study offers compelling evidence for the utility of CRP, D-dimer, and WBC in detecting PJI, additional research is needed to evaluate the cost- effectiveness of using these markers in routine clinical practice. Integrating these markers into a comprehensive diagnostic approach, including clinical assessment and microbiological culture, could improve overall diagnostic accuracy and reduce the risk of false negatives and delays in treatment.

## Conclusion

This study demonstrates that inflammatory markers, including CRP, D-dimer, WBC, sCD14, and SAA, effectively detect periprosthetic joint infection (PJI) in osteoporotic patients undergoing hip arthroplasty. CRP, D-dimer, and WBC were exceptionally reliable, with significant differences observed 48 hours post-surgery. While sCD14 and SAA showed promise, they were less informative than the core markers.

Further research with larger cohorts is needed to confirm these findings and refine the use of these markers for early PJI detection, potentially improving clinical outcomes.

## Dodatak

### Acknowledgements

We sincerely thank the patients who participated in this study for their cooperation and commitment. We also extend our thanks to the medical, nursing, and research staff at the Department of Orthopedics, The Second Affiliated Hospital of Soochow University, and the War and Trauma Emergency Centre, Changhai Hospital, Navy Military Medical University, for their invaluable contributions to patient care and data collection.

### Funding

This study received no funding.

### Author contributions

Conceptualisation: Panfeng Wang, Yongming Sun, Methodology: Panfeng Wang, Dayuan Xu, Yan Xia, Yuntong Zhang, Data Collection: Panfeng Wang, Dayuan Xu, Yuntong Zhang, Data Analysis: Dayuan Xu, Yan Xia, Writing - Original Draft: Panfeng Wang, Dayuan Xu, Yan Xia, Yuntong Zhang, Writing - Review & Editing: Panfeng Wang, Yongming Sun, Supervision: Yongming Sun.

All authors who contributed equally to this work have read and approved the final manuscript. These authors are considered as co-first authors: Panfeng Wang, Dayuan Xu, Yan Xia, and Yuntong Zhang.

Panfeng Wang, Dayuan Xu. These authors contribute to the work equally and are regarded as co-first authors.

### Data availability

The datasets used and analysed during the current study are available from the corresponding author upon reasonable request. Due to privacy regulations and institutional policies, the data are not publicly available.

### Conflict of interest statement

All the authors declare that they have no conflict of interest in this work.
